# Synthesis and Antitumor Activities of Derivatives of the Marine Mangrove Fungal Metabolite Deoxybostrycin

**DOI:** 10.3390/md10122715

**Published:** 2012-11-30

**Authors:** Hong Chen, Xun Zhu, Li-Li Zhong, Bing Yang, Jia Li, Jue-Heng Wu, Sheng-Ping Chen, Yong-Cheng Lin, Yuhua Long, Zhi-Gang She

**Affiliations:** 1 School of Chemistry and Chemical Engineering, Sun Yat-sen University, 135 Xingang West Road, Guangzhou 510275, China; Email: chenwexpo@sina.com (H.C.); zhonglili42@yahoo.com.cn (L.-L.Z.); yangbin_zs@163.com (B.Y.); nuekagami@163.com (J.L.); ceslyc@mail.sysu.edu.cn (Y.-C.L.); 2 Department of Microbiology, Zhongshan School of Medicine, Sun Yat-sen University, 74 Zhongshan Road II, Guangzhou 510080, China; Email: zhuxun8@mail.sysu.edu.cn (X.Z.); wujh@mail.sysu.edu.cn (J.-H.W.); chenshp@mail.sysu.edu.cn (S.-P.C.); 3 Guangdong Province Key Laboratory of Functional Molecules in Oceanic Microorganism, Bureau of Education, Sun Yat-sen University, 74 Zhongshan Road II, Guangzhou 510080, China; 4 School of Chemistry and Environment, South China Normal University, 348 West Outer Ring Road, Guangzhou 510006, China

**Keywords:** deoxybostrycin derivatives, antitumor activity, marine mangrove, anthraquinone

## Abstract

Deoxybostrycin (**1**) is an anthraquinone compound derived from the marine mangrove fungus *Nigrospora* sp. No. 1403 and has potential to be a lead for new drugs because of its various biological properties. A series of new derivatives (**2**–**22**) of deoxybostrycin were synthesized. The *in vitro* cytotoxicity of all the new compounds was tested against MDA-MB-435, HepG2 and HCT-116 cancer cell lines. Most of the compounds exhibit strong cytotoxicity with IC_50_ values ranging from 0.62 to 10 μM. Compounds **19**, **21** display comparable cytotoxicity against MDA-MB-435 to epirubicin, the positive control. The primary screening results indicate that the deoxybostrycin derivatives might be a valuable source of new potent anticancer drug candidates.

## 1. Introduction

Mortality and morbidity of cancer patients is the second highest among all diseases in the world, following heart disease [1,2,3]. Due to drawbacks of chemotherapy, such as dose limits, side effects, and low selectivity to cancer cells, discovery and development of much more effective, safe and highly selective antitumor drugs is still an urgent task.

In recent decades, much effort has been directed toward using natural products as a source of novel anticancer drugs. Recent reviews of drug discovery literature have shown that more than two thirds of the anticancer drugs approved between the 1940s and 2006 are either natural products or developed based on the knowledge gained from natural products [4,5]. In recent years, marine microorganisms have attracted great attention in the pharmaceutical community as they produce a wide variety of metabolites that are structurally unique and pharmacologically active [6,7]. Due to the structural and bioactive diversities of marine microorganism metabolites, they represent a promising resource for discovering new anticancer drugs [8,9]. 

**Figure 1:**
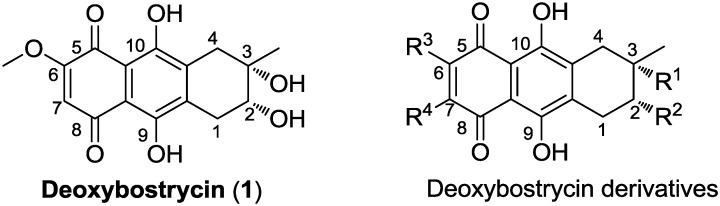
Structures of deoxybostrycin and deoxybostrycin derivatives.

**Scheme 1:**
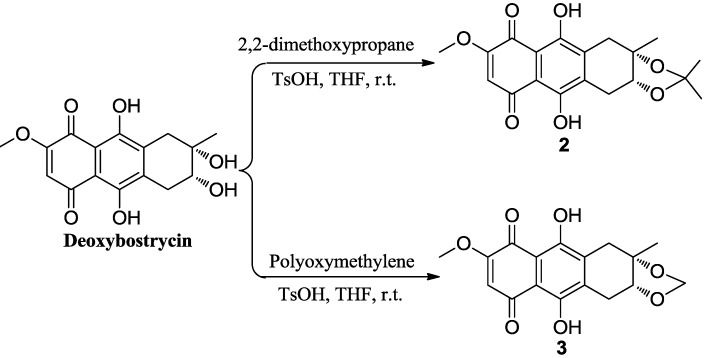
Synthesis of 2,3-ketal deoxybostrycin derivatives **2** and **3**.

**Scheme 2:**
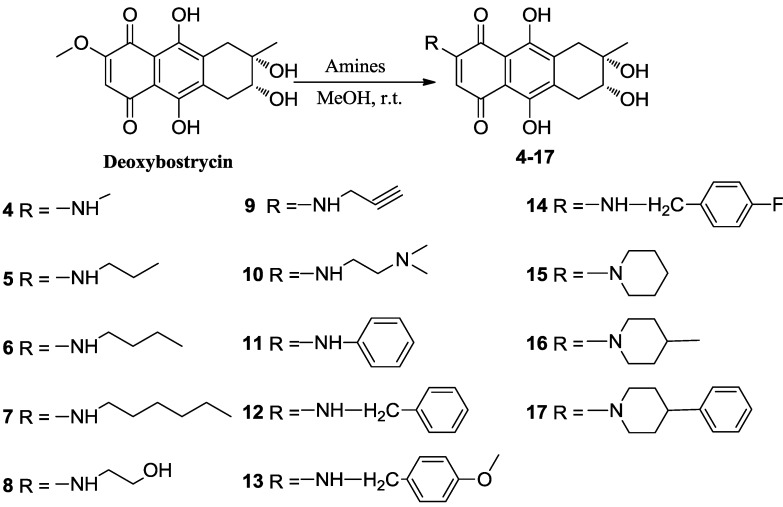
Synthesis of 6-aminosubstituted deoxybostrycin derivatives **4**–**17**.

**Scheme 3:**
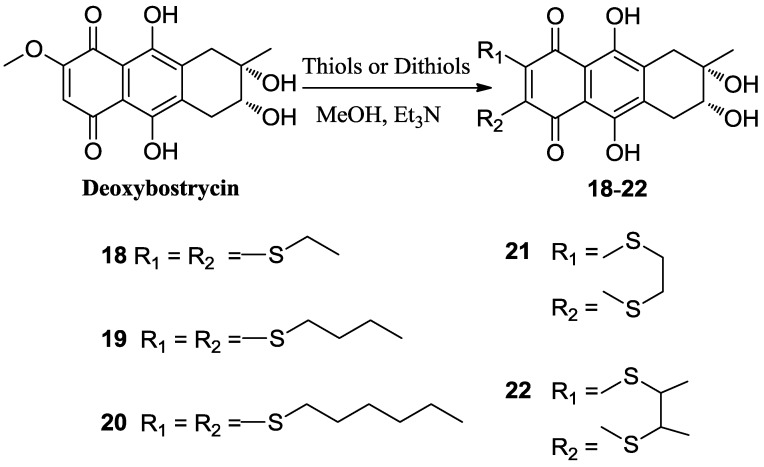
Synthesis of 6,7-dithiosubstituted deoxybostrycin derivatives **18**–**22**.

Deoxybostrycin (**1**, Figure 1), a natural tetrahydroanthraquinone compound, isolated from the mangrove endophytic fungus *Nigrospora* sp. No. 1403 from the South China Sea [10], displays various biological properties including phytotoxic [11], antimalarial [12], antibacterial and cytotoxic activities [10,13]. Its structure was identified by interpretation of spectral data (IR, UV, MS, ^1^H NMR, ^13^C NMR) [11,14,15]. Previous studies demonstrated that deoxybostrycin analogues can affect energy-yielding and energy-requiring processes in Ehrlich ascite cells [16] and inhibit the growth of cultured cells of *Nicotiana rustica*. Additionally, they act as a potent stimulator of NADH oxidation in mitochondria and as electron acceptors in an enzyme preparation of diaphorase [17]. However, there has been no study on the structural modification and anticancer activity of deoxybostrycin. In this paper, we describe the synthesis and cytotoxicity of deoxybostrycin derivatives. A series of deoxybostrycin derivatives (**2**–**22**, Scheme 1, Scheme 2 and Scheme 3) were synthesized by modifying deoxybostrycin at C-2, C-3, C-6 and C-7 positions. All compounds were evaluated for their cytotoxicity against MDA-MB-435, HepG2 and HCT-116 cancer cell lines. Structure-activity relationships were discerned from the cytotoxic experimental data. As we expected, most of the deoxybostrycin derivatives exhibit strong anticancer activities against the tested cancer cell lines. Some of the derivatives showed higher cytotoxicity than the parent compound deoxybostrycin. Compounds **19** and **21** showed comparable cytotoxicity to epirubicin against MDA-MB-435 cell line with IC_50_ values of 0.66 μM and 0.62 μM, respectively.

## 2. Results and Discussion

### 2.1. Chemistry

Deoxybostrycin reacted with 2,2-dimethoxypropane and polyoxymethylene in the presence of 1 equivalent of *p*-toluenesulfonic acid (TsOH) at room temperature to give 2,3-ketal derivatives **2** and **3**, respectively (Scheme 1). When deoxybostrycin reacted with various amines at room temperature using methanol as solvent, a series of alkylamino and arylamino derivatives **4**–**17** were obtained (Scheme 2). Dithiosubstitued derivatives **18**–**22** were afforded by the reaction of deoxybostrycin with various thiols and dithiols at 0–5 °C in the presence of triethylamine (Scheme 3). The detailed mechanism of the nucleophilic substitution reaction of deoxybostrycin with thiols was proposed in our previous work [15]. The structures of all the synthesized compounds were characterized by IR, ^1^H NMR, ^13^C NMR and HRMS (ESI).

### 2.2. Biological Activity

All synthesized compounds were evaluated for their *in vitro* cytotoxic activity against three human cancer cell lines (MDA-MB-435, HepG2 and HCT-116) by microculture tetrazolium assay (MTT) assay [18] using epirubicin as positive control.

As shown in Table 1, most of the deoxybostrycin derivatives showed good to excellent cytotoxic activity against the three tested cancer cell lines with IC_50_ < 10 μM. Some modified compounds exhibited better antitumor activities than the parent compound deoxybostrycin, and even displayed comparable activity to epirubicin. For example, the activity of compound **19** against MDA-MB-435 cell line (IC_50_ = 0.66 μM) showed comparable activity to epirubicin (IC_50_ = 0.56 μM). Similar potency was observed with compound **21** (against MDA-MB-435 and HCT-116 cells) and **22** (against MDA-MB-435 cells). Moreover, compounds **9**, **11**, **12**, **15**, **16** and **22** exhibited selectivity for MDA-MB-435 over other cell lines. The cytotoxic activities of compounds **6**, **13**, **14**, **18** and **20** against MDA-MB-435 and HCT-116 cell lines were stronger than against HepG2 cell line. Compound **21** possessed the most potent activity against HCT-116 cell lines with an IC_50_value of 0.80 μM. Some results can be concluded from the SAR (structure-activity relationships) analysis based on the cytotoxic data of deoxybostrycin and its derivatives: (1) ketal **2** and **3** exhibited lower cytotoxic activities against all tested cancer cell lines than that of deoxybostrycin. The results suggested that the hydroxyl at C-2 and C-3 of deoxybostrycin was favorable for antitumor activity. Transformation of the diol to the diether decreased activity. Compound **2** with high steric hindrance at C-2 and C-3 showed nearly no cellular cytotoxic activity; (2) Compounds **4**–**17** derived from the replacement of methoxyl with various amines at the C-6 position generally decreased the cellular cytotoxicity with respect to the parent compound. The cytotoxic activity of Compounds **4**–**6** against HCT-116 cell lines showed alkylamino chain length dependence. The IC_50_ values change from about 16 μM to 3 μM with the chain length increasing from one carbon for methylamine to six carbons for hexamine. Although compounds **9**, **11**, **12**, **15** and **16** displayed decreased potency against HepG2 and HCT-116 cell lines, they had significantly improved selectivity for MDA-MB-435 cell. Compound **10** with a *N*,*N*-Dimethylethylenediamine substituent exhibited better cytotoxic activity against HepG2 cells than all other amino-substituted derivatives. Compared to benzylamino derivative **12**, *p*-methoxy and *p*-fluorine substituted benzylamino derivatives **13** and **14** showed higher cytotoxic activity against HCT-116 cell line; (3) Compounds **18**–**22** were alkylthio-substituted derivatives of deoxybostrycin at C-6 and C-7 positions. All the dialkylthio-substituted deoxybostrycin derivatives showed excellent cytotoxic activity against all three human cancer cell lines with IC_50_ values between 0.62 μM and 6.49 μM except for compounds **18** and **20** against HepG2 cell line. Among all the derivatives, compound **21** characterized with a relatively rigid 2,3-dihydro-1,4-dithiine heterocycle attached to deoxybostrycin displayed the highest potency against all the three tested cancer cell lines. Significantly, compound **21** displayed a comparable cytotoxic activity with the positive control epirubicin, for instance, compound **21** against MDA-MB-435 cell with an IC_50_ of 0.62 μM *vs.* epirubicin against MDA-MB-435 cell with an IC_50_ of 0.56μM. The results suggest that the dithio-substituted deoxybostrycin derivatives benefit cytotoxic activity and serve as promising scaffolds for anti-tumor agents. These positive results serve as a valuable guideline for further research on the structural optimization, mechanism study and development of deoxybostrycin derivatives as novel anti-tumor agents.

**Table 1:** Cytotoxicity(IC_50_, μM) of compounds **1**–**22** against MDA-MB-435, HepG2 and HT-116 cancer cell lines.

Compounds		IC_50_ (μM) ^a^	
	MDA-MB-435 ^b^	HepG2 ^b^	HCT-116 ^b^
**1**	3.19 ± 0.92	9.99 ± 0.55	5.69 ± 0.25
**2**	>50	>50	26.08 ± 1.84
**3**	3.06 ± 0.13	12.83 ± 0.15	7.55 ± 0.45
**4**	11.74 ± 1.12	>50	16.57 ± 1.40
**5**	>50	9.98 ± 1.06	7.54 ± 0.21
**6**	6.79 ± 1.59	>50	3.29 ± 0.01
**7**	10.00 ± 1.75	>50	3.14 ± 0.16
**8**	>50	>50	>50
**9**	6.31 ± 1.40	10.90 ± 1.40	25.79 ± 0.64
**10**	1.52 ± 0.72	2.26 ± 0.35	3.42 ± 0.21
**11**	9.67 ± 1.80	>50	>50
**12**	5.76 ± 2.75	13.37 ± 2.72	20.70 ± 2.76
**13**	5.81 ± 2.89	10.09 ± 0.82	9.62 ± 0.20
**14**	7.25 ± 2.27	11.18 ± 0.94	7.09 ± 0.09
**15**	7.33 ± 1.20	>50	16.34 ± 0.90
**16**	7.32 ± 1.04	>50	14.92 ± 1.02
**17**	>50	11.81 ± 1.45	20.64 ± 1.64
**18**	1.96 ± 0.58	11.56 ± 1.40	6.49 ± 0.73
**19**	0.66 ± 0.41	5.04 ± 1.38	2.75 ± 0.23
**20**	2.06 ± 0.17	>50	2.94 ± 0.10
**21**	0.62 ± 0.23	1.98 ± 0.34	0.80 ± 0.08
**22**	0.97 ± 0.24	2.39 ± 0.50	2.69 ± 0.23
Epirubicin ^c^	0.56 ± 0.06	0.96 ± 0.02	0.48 ± 0.03

^a^ IC_50_ values are taken as means ± standard deviation from three independent experiments; ^b^ MDA-MB-435, human breast cancer cell line; HepG2, human liver cancer cell line; HCT-116, human colon cancer cell line; ^c^ Used as a positive control.

## 3. Experimental Section

### 3.1. Chemistry

Reagents were commercially available and used as received. Solvents were dried and purified using standard techniques. Melting points were measured on an X-4 micromelting point apparatus and were uncorrected. IR spectra were measured on a Bruker Vector 22 spectrophotometer using KBr pellets. NMR spectra were determined on a Varian Mercury-Plus 300 spectrometer or Bruker AV-400 NB spectrometer in CDCl_3_ or DMSO-*d*_6_ using TMS as internal standard, and coupling constants (*J*) are in Hz. ESI mass spectra were obtained on a LCQ DECA XP LC-MS mass spectrometer. Flash column chromatography was run on silica gel (Qing dao Ocean Chemical Factory, 200–300 mesh) eluted with petroleum ether-dichloromethane or dichloromethane-methanol, and C18 reversed phase silica gel (Welch Material, Inc., 45 μm) eluted with methanol-water.

### 3.2. Synthesis of 2,3-*O*-(isopropylidene) Deoxybostrycin (**2**)

To a solution of **1** (50 mg, 0.156 mmol) in 10 mL of tetrahydrofuran were added 2,2-dimethoxypropane (323.4 mg, 3.13 mmol) and *p*-toluenesulfonic acid (26.8 mg, 0.156 mmol). The reaction mixture was stirred for 15 h at room temperature and then diluted with water (20 mL) and extracted with dichloromethane (3 × 50 mL). The combined organic layer was washed with brine, dried over anhydrous magnesium sulfate, and concentrated *in vacuo*. The resulting residue was purified on a silica gel column using petroleum ether/dichloromethane (v/v, 1/1) as eluent to obtain 48.3 mg of compound **2** as a red solid in an 86% yield. Mp: 160–161 °C; [α]^20^_D_ = 27.0° (*c* = 1.00, CH_3_OH); IR (KBr): ν_max_ = 3431, 3086, 2980, 2931, 2896, 2883, 1598, 1564, 1452, 1415 cm^−1^; ^1^H NMR (300 MHz, CDCl_3_): δ 13.12 (s, 1H), 12.71 (s, 1H), 6.17 (s, 1H), 4.39 (dd, 1H, *J* = 3.8, 2.9 Hz), 3.93 (s, 3H), 3.53 (dd, 1H, *J* = 16.7, 2.9 Hz), 3.33 (d, 1H, *J* = 16.1 Hz), 2.54 (dd, 1H, *J* = 16.7, 3.8 Hz), 2.33 (d, 1H, *J* = 16.1 Hz), 1.51 (s, 3H), 1.36 (s, 3H), 1.07 (s, 3H); ^13^C NMR (75 MHz, CDCl_3_): δ 185.92, 179.69, 160.84, 159.33, 158.09, 139.05, 137.04, 110.04, 109.98, 108.47, 80.89, 79.38, 57.05, 33.97, 27.94, 27.58, 27.13, 26.86; ESI-MS *m/z*: 359.2 [M − H]^−^;HRMS (EI) calcd for C_19_H_20_O_7_, 360.3579; found, 360.1207.

### 3.3. Synthesis of 2,3-*O*-(methylene) Deoxybostrycin (**3**)

To a solution of **1** (100 mg, 0.313 mmol) in 10 mL of tetrahydrofuran were added polyoxymethylene (45 mg) and *p*-toluenesulfonic acid (53.8 mg, 0.298 mmol). The reaction mixture was stirred for 20 h at room temperature and then with water (20 mL) and extracted with dichloromethane (3 × 50 mL). The combined organic layer was washed with brine, dried over anhydrous magnesium sulfate, and concentrated *in vacuo*. The resulting residue was purified on a silica gel column using dichloromethane-methanol (v/v, 250/1) as eluent to obtain 54 mg compound **3** as a red solid (CH_2_Cl_2_) in a 52% yield. Mp:150–152 °C; IR (KBr): ν_max_ = 3433, 3072, 2967, 2953, 2908, 2886, 1594, 1479, 1444, 1420 cm^−1^; ^1^H NMR (300 MHz, CDCl_3_): δ 13.12 (s, 1H), 12.70 (s, 1H), 6.16 (s, 1H), 4.82 (s, 1H), 4.73 (s, 1H), 4.15 (dd, 1H, *J* = 4.0, 3.0 Hz), 3.93 (s, 3H), 3.58 (dd, 1H, *J* = 16.5, 3.0 Hz), 3.48 (d, 1H, *J* = 16.1 Hz), 2.51 (dd, 1H, *J* = 16.5, 4.0 Hz), 2.35 (d, 1H, *J* = 16.1 Hz), 1.47 (s, 3H); ^13^C NMR (75 MHz, CDCl_3_): δ 189.14, 186.20, 160.84, 158.97, 157.61, 138.48, 136.51, 110.32, 109.98, 108.80, 79.77, 79.62, 57.06, 32.34, 26.58, 24.97; ESI-MS *m/z*: 331.0 [M − H]^−^.

### 3.4. General Procedure for Preparation of Compounds (**4–17**)

To a solution of **1 **(50 mg, 0.156 mmol) in 10 mL of methanol was added the corresponding amine (0.78 mmol). The reaction mixture was stirred at room temperature until the starting material disappeared (for aniline, the reaction mixture was stirred at 50 °C). The solvent was removed under reduced pressure. The resulting residue was subsequently purified using first silica gel chromatography with dichloromethane-methanol as eluent, then C18 reversed phase silica gel chromatography with methanol-water as eluent.

#### 3.4.1. 6-(Methylamino) 1-Deoxy-6-demethoxybostrycin (**4**)

A red solid (MeOH) in a 40% yield; mp: 221–223 °C; IR (KBr): ν_max_ = 3375, 3338, 2929, 2901, 2853, 2814, 1582, 1514, 1452, 1418 cm^−1^; ^1^H NMR (400 MHz, DMSO-*d*_6_): δ 14.23 (s, 1H), 12.36 (s, 1H), 7.95 (q, 1H, *J* = 5.0 Hz), 5.55 (s, 1H), 4.75 (d, 1H, *J* = 5.1 Hz), 4.41 (s, 1H), 3.63 (dt, 1H, *J* = 7.3, 5.1 Hz), 2.83 (d, 3H, *J* = 5.0 Hz), 2.85 (dd, 1H, *J* = 18.8, 5.1 Hz), 2.77 (d, 1H, *J* = 17.9 Hz), 2.68 (dd, 1H, *J* = 18.8, 7.3 Hz), 2.57 (d, 1H, *J* = 17.9 Hz), 1.20 (s, 3H); ^13^C NMR (100 MHz, DMSO-*d*_6_): δ 186.31, 183.47, 156.34, 154.56, 151.05, 139.24, 132.89, 109.17, 107.89, 98.98, 70.74, 69.38, 35.99, 30.70, 29.62, 25.93; ESI-MS *m/z*: 318.1 [M − H]^−^; HRMS (EI) calcd for C_16_H_17_NO_6_, 319.1056; found, 319.1059.

#### 3.4.2. 6-(*n*-Propylamino) 1-Deoxy-6-demethoxybostrycin (**5**)

A red solid (MeOH) in a 44% yield; mp: 215–216 °C; IR (KBr): ν_max_ = 3385, 3285, 3085, 2966, 2934, 2876, 1578, 1508, 1445, 1412 cm^−1^; ^1^H NMR (400 MHz, DMSO-*d*_6_): δ 14.23 (s, 1H), 12.37 (s, 1H), 7.86 (t, 1H, *J* = 5.7 Hz), 5.63 (s, 1H), 4.74 (d, 1H, *J* = 5.1 Hz), 4.40 (s, 1H), 3.63 (dt, 1H, *J* = 7.3, 5.1 Hz), 3.18 (dt, 2H, *J* = 5.7, 7.2 Hz), 2.85 (1H, dd, *J* = 18.7, 5.1 Hz), 2.79 (d, 1H, *J* = 17.9 Hz), 2.68 (dd, 1H, *J* = 18.7, 7.3 Hz), 2.57 (d, 1H, *J* = 17.9 Hz), 1.61 (sextet, 2H, *J* = 7.2 Hz), 1.20 (s, 3H), 0.91 (t, 3H, *J* = 7.4 Hz); ^13^C NMR (100 MHz, DMSO-*d*_6_): δ 186.34, 183.54, 156.35, 154.50, 150.14, 139.28, 132.84, 109.18, 107.79, 98.96, 70.74, 69.37, 44.26, 36.00, 30.69, 25.93, 21.22, 11.82; ESI-MS *m/z*: 346.2 [M − H]^−^; HRMS (EI) calcd for C_18_H_21_NO_6_, 347.1369; found, 347.1370.

#### 3.4.3. 6-(*n*-Butylamino) 1-Deoxy-6-demethoxybostrycin (**6**)

A red solid (MeOH) in a 40% yield; mp: 215–217 °C; IR (KBr): ν_max_ = 3386, 3287, 3085, 2960, 2934, 2872, 1580, 1509, 1445, 1412 cm^–1^; ^1^H NMR (400 MHz, DMSO-*d*_6_): δ 14.22 (s, 1H), 12.36 (s, 1H), 7.85 (t, 1H, *J* = 6.0 Hz), 5.61 (s, 1H), 4.74 (d, 1H, *J* = 5.1 Hz), 4.40 (s, 1H), 3.63 (dt, 1H, *J* = 7.4, 5.2 Hz), 3.21 (dt, 2H, *J* = 6.0, 7.1 Hz), 2.85 (dd, 1H, *J* = 18.7, 5.2 Hz), 2.79 (d, 1H, *J* = 17.9 Hz), 2.68 (dd, 1H, *J* = 18.7, 7.4 Hz), 2.57 (d, 1H, *J* = 17.9 Hz), 1.57 (pentet, 2H, *J* = 7.1 Hz), 1.35 (sextet, 2H, *J* = 7.3 Hz), 1.20 (s, 3H), 0.91 (t, 3H, *J* = 7.3 Hz); ^13^C NMR (100 MHz, DMSO-*d*_6_): δ 186.30, 183.53, 156.35, 154.50, 150.09, 139.29, 132.83, 109.18, 107.79, 98.91, 70.74, 69.37, 42.33, 36.00, 30.68, 29.90, 25.94, 20.16, 14.13; ESI-MS m/z: 360.2 [M − H]^−^; HRMS (EI) calcd for C_19_H_23_NO_6_, 361.1525; found, 361.1522.

#### 3.4.4. 6-(*n*-Hexylamino) 1-Deoxy-6-demethoxybostrycin (**7**)

A red solid (MeOH) in a 52% yield; mp: 214–215 °C; IR (KBr): ν_max_ = 3384, 3287, 3086, 2955, 2932, 2870, 2858, 1576, 1509, 1448, 1410 cm^−1^; ^1^H NMR (400 MHz, DMSO-*d*_6_): δ 14.25 (s, 1H), 12.38 (s, 1H), 7.88 (t, 1H, *J* = 6.1 Hz), 5.63 (s, 1H), 4.75 (d, 1H, *J* = 5.1 Hz), 4.41 (s, 1H), 3.64 (dt, 1H, *J* = 7.2, 5.2 Hz), 3.21 (dt, 2H, *J* = 6.1, 7.0 Hz), 2.86 (dd, 1H, *J* = 18.7, 5.2 Hz), 2.80 (d, 1H, *J* = 18.0 Hz), 2.69 (dd, 1H, *J* = 18.7, 7.2 Hz), 2.58 (d, 1H, *J* = 18.0 Hz), 1.57 (m, 2H), 1.37–1.25 (m, 6H), 1.19 (s, 3H), 0.87 (t, 3H, *J* = 6.8 Hz); ^13^C NMR (100 MHz, DMSO-*d*_6_): δ 186.35, 183.58, 156.36, 154.51, 150.13, 139.28, 132.88, 109.21, 107.82, 98.90, 70.73, 69.38, 42.61, 35.97, 31.39, 30.71, 27.75, 26.59, 25.91, 22.49, 14.35; ESI-MS *m/z*: 388.1 [M − H]^−^; HRMS (EI) calcd for C_21_H_27_NO_6_, 389.1838; found, 389.1834.

#### 3.4.5. 6-(2′-Hydroxyethylamino) 1-Deoxy-6-demethoxybostrycin (**8**)

A red solid (MeOH) in a 30% yield; mp: 232–234 °C; IR (KBr): ν_max_ = 3441, 3389, 3340, 3298, 3062, 2966, 2945, 2926, 2875, 1571, 1528, 1445, 1419 cm^−1^; ^1^H NMR (400 MHz, DMSO-*d*_6_): δ 14.21 (s, 1H), 12.36 (s, 1H), 7.63 (t, 1H, *J* = 5.9 Hz), 5.70 (s, 1H), 4.89 (br t, 1H), 4.75 (d, 1H, *J* = 4.2 Hz), 4.41 (s, 1H), 3.66–3.59 (m, 3H), 3.28 (q, 2H, *J* = 5.9 Hz), 2.86 (dd, 1H, *J* = 18.7, 5.0 Hz), 2.80 (d, 1H, *J* = 18.0 Hz), 2.69 (dd, 1H, *J* = 18.7, 7.4 Hz), 2.58 (d, 1H, *J* = 18.0 Hz), 1.20 (s, 3H); ^13^C NMR (100 MHz, DMSO-*d*_6_): δ 186.46, 183.39, 156.43, 154.57, 150.35, 139.33, 132.99, 109.12, 107.78, 99.37, 70.73, 69.38, 58.95, 45.34, 36.01, 30.70, 25.92; ESI-MS *m/z*: 348.1 [M − H]^−^; HRMS (EI) calcd for C_17_H_19_NO_7_, 349.1162; found, 349.1158.

#### 3.4.6. 6-(Prop-2′-yn-1′-ylamino) 1-Deoxy-6-demethoxybostrycin (**9**)

A red solid (MeOH) in a 56% yield; mp: 214–216 °C; IR (KBr): ν_max_ = 3390, 3242, 3242, 3070, 2968, 2932, 2912, 2851, 1586, 1503, 1447, 1387 cm^−1^; ^1^H NMR (400 MHz, DMSO-*d*_6_): δ 14.03 (s, 1H), 12.36 (s, 1H), 8.08 (t, 1H, *J* = 5.8 Hz), 5.74 (s, 1H), 4.76 (d, 1H, *J* = 5.1 Hz), 4.42 (s, 1H), 4.08 (dd, 2H, *J* = 5.8, 2.3 Hz), 3.64 (dt, 1H, *J* = 7.3, 5.2 Hz), 3.27 (t, 1H, *J* = 2.3 Hz), 2.86 (dd, 1H, *J* = 18.8, 5.1 Hz), 2.80 (d, 1H, *J* = 18.0 Hz), 2.69 (dd, 1H, *J* = 18.8, 7.3 Hz), 2.59 (d, 1H, *J* = 18.0 Hz), 1.20 (s, 3H); ^13^C NMR (100 MHz, DMSO-*d*_6_): δ 186.54, 183.10, 156.66, 154.94, 149.46, 139.31, 133.53, 109.10, 107.64, 101.23, 79.13, 75.25, 70.71, 69.36, 36.05, 31.83, 30.67, 25.92; ESI-MS *m/z*: 342.1 [M − H]^−^; HRMS (EI) calcd for C_18_H_1__7_NO_6_, 343.1056; found, 343.1052.

#### 3.4.7. 6-[(2′-(Dimethylamino)ethyl)amino]-1-deoxy-6-demethoxybostrycin (**10**)

A red solid (MeOH) in a 60% yield; mp: 215–217 °C; IR (KBr): ν_max_ = 3287, 3087, 2974, 2944, 2863, 2818, 1575, 1512, 1444, 1411 cm^−1^; ^1^H NMR (400 MHz, DMSO-*d*_6_): δ 14.19 (s, 1H), 12.29 (s, 1H), 7.47 (t, 1H, *J* = 5.4 Hz), 5.67 (s, 1H), 4.75 (d, 1H, *J* = 4.6 Hz), 4.41 (s, 1H), 3.63 (dt, 1H, *J* = 7.4, 5.3 Hz), 3.37–3.26 (m, 4H), 2.86 (dd, 1H, *J* = 18.7, 5.2 Hz), 2.79 (d, 1H, *J* = 18.1 Hz), 2.68 (dd, 1H, *J* = 18.7, 7.4 Hz), 2.59 (d, 1H, *J* = 18.1 Hz), 2.25 (s, 6H), 1.20 (s, 3H); ^13^C NMR (100 MHz, DMSO-*d*_6_): δ 186.44, 183.21, 156.45, 154.65, 149.81, 139.40, 133.07, 109.10, 107.78, 99.47, 70.73, 69.37, 56.39, 45.28, 36.01, 30.70, 25.92; ESI-MS *m/z*: 375.1 [M − H]^−^; HRMS (EI) calcd for C_19_H_24_N_2_O_6_, 376.1634; found, 376.1628.

#### 3.4.8. 6-(Phenylamino) 1-Deoxy-6-demethoxybostrycin (**11**)

A red solid (MeOH) in a 38% yield; mp: 220–222 °C; IR (KBr): ν_max_ = 3359, 3283, 3060, 2980, 2940, 2857, 2821, 1584, 1543, 1444, 1382 cm^−1^; ^1^H NMR (400 MHz, DMSO-*d*_6_): δ 13.92 (s, 1H), 12.49 (s, 1H), 9.46 (s, 1H), 7.48–7.24 (m, 5H), 6.00 (s, 1H), 4.76 (br s, 1H), 4.43 (s, 1H), 3.64 (br t, 1H), 2.86 (dd, 1H, *J* = 18.6, 5.0 Hz), 2.82 (d, 1H, *J* = 18.2 Hz), 2.69 (dd, 1H, *J* = 18.8, 7.4 Hz), 2.60 (d, 1H, *J* = 18.2 Hz), 1.20 (s, 3H); ^13^C NMR (100 MHz, DMSO-*d*_6_): δ 186.93, 183.08, 156.96, 154.94, 147.89, 139.26, 138.24, 133.89, 129.83,126.18, 124.48,109.21, 107.80, 101.70, 70.72, 69.38, 36.14, 30.66, 25.93; ESI-MS *m/z*: 380.1 [M − H]^−^; HRMS (EI) calcd for C_21_H_19_NO_6_, 381.1212; found, 381.1204.

#### 3.4.9. 6-(Benzylamino) 1-Deoxy-6-demethoxybostrycin (**12**)

A red solid (MeOH) in 29% yield; mp: 220–222 °C; IR (KBr): ν_max_ = 3382, 3264, 3085, 2973, 2934, 2900, 2870, 1574, 1511, 1444, 1411 cm^−1^; ^1^H NMR (300 MHz, DMSO-*d*_6_): δ 12.37 (s, 1H), 8.49 (t, 1H, *J* = 6.4 Hz), 7.38–7.18 (m, 5H), 5.53 (s, 1H), 4.75 (d, 1H, *J* = 5.3 Hz), 4.46 (d, 2H, *J* = 6.4 Hz), 4.42 (s, 1H), 3.61 (dt, 1H, *J* = 7.5, 5.3 Hz), 2.82 (dd,1H, *J* = 18.9, 5.3 Hz), 2.77 (d, 1H, *J* = 17.4 Hz), 2.66 (dd, 1H, *J* = 18.9, 7.5 Hz), 2.57 (d, 1H, *J* = 17.4 Hz,), 1.17 (s, 3H); ESI-MS *m/z*: 394.1 [M − H]^−^; HRMS (EI) calcd for C_22_H_21_NO_6_, 395.1369; found, 395.1361.

#### 3.4.10. 6-(*p*-Methoxybenzylamino) 1-Deoxy-6-demethoxybostrycin (**13**)

A red solid (MeOH) in a 44% yield; mp: 223–225 °C; [α]^20^_D_ = −250.0° (*c* = 1.00, CH_3_OH); IR (KBr): ν_max_ = 3373, 3069, 2979, 2938, 2861, 2834, 1583, 1507, 1445, 1387 cm^–1^; ^1^H NMR (400 MHz, DMSO-*d*_6_): δ 14.11 (s, 1H), 12.39 (s, 1H), 8.40 (t, 1H, *J* = 6.5 Hz), 7.33–6.87 (m, 4H), 5.57 (s, 1H), 4.74 (d, 1H, *J* = 5.1 Hz), 4.40 (br s, 2H), 4.39 (s, 1H), 3.73 (s, 3H), 3.63 (dt, 1H, *J* = 7.2, 5.2 Hz), 2.84 (dd, 1H, *J* = 18.8, 5.2 Hz), 2.80 (d, 1H, *J* = 18.1 Hz), 2.68 (dd, 1H, *J* = 18.8, 7.2 Hz), 2.58 (d, 1H, *J* = 18.1 Hz), 1.19 (s, 3H); ^13^C NMR (100 MHz, DMSO-*d*_6_): δ 186.33, 183.51, 158.95, 156.45, 154.65, 149.93, 139.26, 133.12, 129.42, 129.04, 129.04, 114.42, 114.42, 109.18, 107.72, 100.10, 70.71, 69.36, 55.52, 45.25, 35.99, 30.68, 25.91; ESI-MS *m/z*: 424.1 [M − H] ^−^; HRMS (EI) calcd for C_23_H_23_NO_7_, 425.1475; found, 425.1464.

#### 3.4.11. 6-(*p*-Fluorobenzylamino) 1-Deoxy-6-demethoxybostrycin (**14**)

A red solid (MeOH) in a 47% yield; mp: 234–236 °C; IR (KBr): ν_max_ = 3272, 3085, 2976, 2936, 2873, 2856, 1577, 1510, 1443, 1411 cm^−1^; ^1^H NMR (400 MHz, DMSO-*d*_6_): δ 14.07 (s, 1H), 12.40 (s, 1H), 8.46 (t, 1H, *J* = 6.5 Hz), 7.44–7.14 (m, 4H), 5.57 (s, 1H), 4.74 (d, 1H, *J* = 5.1 Hz), 4.46 (d, 2H, *J* = 6.5 Hz), 4.41 (s, 1H), 3.63 (dt, 1H, *J* = 7.3, 5.2 Hz), 2.85 (dd, 1H, *J* = 18.8, 5.2 Hz), 2.80 (d, 1H, *J* = 18.1 Hz), 2.68 (dd, 1H, *J* = 18.8, 7.2 Hz), 2.58 (d, 1H, *J* = 18.1 Hz), 1.19 (s, 3H); ^13^C NMR (100 MHz, DMSO-*d*_6_): δ 186.43, 183.44, 160.63, 156.47, 154.68, 149.98, 139.24, 133.80, 133.20, 129.77, 129.69, 115.74, 109.19, 107.72, 100.21, 70.71, 69.36, 45.00, 35.99, 30.68, 25.90; ESI-MS *m/z*: 412.1 [M − H]^−^; HRMS (EI) calcd for C_22_H_20_FNO_6_, 413.1275; found, 413.1267.

#### 3.4.12. 6-(Piperidin-1-yl) 1-Deoxy-6-demethoxybostrycin (**15**)

A red solid (MeOH) in a 30% yield; mp: 188–190 °C; IR (KBr): ν_max_ = 3369, 3065, 2935, 2856, 1593, 1551, 1448, 1409 cm^−1^; ^1^H NMR (400 MHz, DMSO-*d*_6_): δ 13.86 (s, 1H), 12.66 (s, 1H), 5.98 (s, 1H), 4.74 (d, 1H, *J* = 5.1 Hz), 4.39 (s, 1H), 3.63 (dt, 1H, *J* = 7.3, 5.2 Hz), 3.55 (br s, 4H), 2.83 (dd, 1H, *J* = 18.7, 5.2 Hz), 2.79 (d, 1H, *J* = 18.2 Hz), 2.67 (dd, 1H, *J* = 18.7, 7.3 Hz), 2.57 (d, 1H, *J* = 18.2 Hz), 1.65 (br s, 6H), 1.19 (s, 3H); ^13^C NMR (100 MHz, DMSO-*d*_6_): δ 185.37, 184.10, 157.65, 155.43, 154.35, 138.14, 134.29, 110.61, 109.80, 107.87, 70.72, 69.40, 50.89,36.22, 30.49, 25.93, 24.15; ESI-MS *m/z*: 372.1 [M − H]^−^; HRMS (EI) calcd for C_20_H_23_NO_6_, 373.1525; found, 373.1522.

#### 3.4.13. 6-(4′-Methylpiperidin-1-yl) 1-Deoxy-6-demethoxybostrycin (**16**)

A red solid (MeOH) in a 32% yield; mp: 198–200 °C; [α]^20^_D_ = −44.8° (*c* = 1.00, CH_3_OH); IR (KBr): ν_max_ = 3407, 3087, 2948, 2923, 2872, 1593, 1573, 1543, 1455 cm^−1^; ^1^H NMR (400 MHz, DMSO-*d*_6_): δ 13.85 (s, 1H), 12.66 (s, 1H), 5.98 (s, 1H), 4.74 (d, 1H, *J* = 5.1 Hz), 4.39 (s, 1H), 4.09 (br d, 2H), 3.63 (dt, 1H, *J* = 7.3, 5.2 Hz), 3.01 (br t, 2H), 2.81 (dd, 1H, *J* = 18.7, 5.2 Hz), 2.79 (d, 1H, *J* = 18.2 Hz), 2.67 (dd, 1H, *J* = 18.7, 7.3 Hz), 2.56 (d, 1H, *J* = 18.2 Hz), 1.75 (m, 2H), 1.67 (m, 1H), 1.31 (m, 2H), 1.19 (s, 3H), 0.94 (d, 3H, *J* = 6.3 Hz); ^13^C NMR (100 MHz, DMSO-*d*_6_): δ 185.35, 184.07, 157.69, 155.47, 154.27, 138.16, 134.30, 110.59, 110.00, 107.86, 70.72, 69.40, 50.14, 36.22, 34.08, 30.50, 25.94; ESI-MS *m/z*: 386.1 [M − H]^−^; HRMS (EI) calcd for C_2__1_H_2__5_NO_6_, 387.1682; found, 387.1678.

#### 3.4.14. 6-(4′-Phenylpiperidin-1-yl) 1-Deoxy-6-demethoxybostrycin (**17**)

A red solid (MeOH) in a 28% yield; mp: 227–228 °C; IR (KBr): ν_max_ = 3332, 3084, 2939, 2927, 2876, 2847, 1593, 1567, 1537, 1454, 1407 cm^−1^; ^1^H NMR (400 MHz, DMSO-*d*_6_): δ 13.85 (s, 1H), 12.70 (s, 1H), 7.36–7.17 (m, 5H), 6.07 (s, 1H), 4.75 (d, 1H, *J* = 5.1 Hz), 4.41 (s, 1H), 4.27 (br d, 2H), 3.64 (dt, 1H, *J* = 7.3, 5.2 Hz), 3.14 (br t, 2H), 2.87 (m, 2H), 2.79 (d, 1H, *J* = 18.2 Hz,), 2.69 (dd, 1H, *J* = 18.7, 7.2 Hz), 2.58 (d, 1H, *J* = 18.2 Hz), 1.81 (m, 4H), 1.20 (s, 3H); ^13^C NMR (100 MHz, DMSO-*d*_6_): δ 185.38, 183.96, 157.85, 155.65, 154.29, 145.87, 138.20, 134.46, 128.90,127.19, 127.19,126.71, 110.63, 110.37, 107.91, 70.72, 69.41, 50.50,41.85, 36.24, 33.23,30.50, 25.94; ESI-MS *m/z*: 448.2 [M − H]^−^; HRMS (EI) calcd for C_2__6_H_2__7_NO_6_, 449.1838; found, 449.1837.

### 3.5. General Procedure for Preparation of Compounds (**18–22**)

To a solution of **1** (50 mg, 0.156 mmol) and triethylamine (8 equivalents) in 10 mL of methanol was added the corresponding thiol (0.624 mmol, the butane-2,3-dithiol was racemate). The reaction mixture was stirred at 0–5 °C until the starting material disappeared. The solvent was removed under reduced pressure. The resulting residue was subsequently purified using first silica gel chromatography with dichloromethane-methanol as eluent, and then C18 reversed phase silica gel column with methanol-water as eluent to obtain the corresponding products.

#### 3.5.1. 6,7-Bis(ethylthio) 1-Deoxy-6-demethoxybostrycin (**18**)

A red solid (MeOH) in a 35% yield; mp: 186–188 °C; IR (KBr): ν_max_ = 3378, 2976, 2959, 2925, 2855, 1611, 1574, 1489, 1427, 1408 cm^−1^; ^1^H NMR (400 MHz, DMSO-*d*_6_): δ 13.11 (s, 2H), 4.79 (br s, 1H), 4.46 (s, 1H), 3.63 (br t, 1H), 3.26 (q, 4H, *J* = 7.4 Hz), 2.83 (dd, 1H, *J* = 18.1, 5.4 Hz), 2.79 (d, 1H, *J* = 18.7 Hz), 2.66 (dd, 1H, *J* = 19.0, 7.4 Hz), 2.58 (d, 1H, *J* = 18.7 Hz), 1.21 (m, 9H); ^13^C NMR (100 MHz, DMSO-*d*_6_): δ 175.21, 165.23, 165.19, 145.47, 138.77, 138.58, 109.40, 70.60, 69.34, 36.27, 30.32, 29.26,25.81, 15.63; ESI-MS *m/z*: 409.0 [M − H]^−^; HRMS (EI) calcd for C_19_H_22_O_6_S_2_, 410.0858; found, 410.0855.

#### 3.5.2. 6,7-Bis(*n*-butylthio) 1-Deoxy-6-demethoxybostrycin (**19**)

A red solid (MeOH) in a 39% yield; mp: 178–180 °C; [α]^20^_D_ = −17.4° (*c* = 1.00, CH_3_OH); IR (KBr): ν_max_ = 3317, 2958, 2929, 2870, 1599, 1438, 1422, 1408 cm^−1^; ^1^H NMR (400 MHz, DMSO-*d*_6_): δ 13.12 (s, 2H), 4.80 (br s, 1H), 4.46 (s, 1H), 3.63 (br t, 1H), 3.25 (t, 4H, *J* = 7.2 Hz), 2.83 (dd, 1H, *J* = 19.0, 5.6 Hz), 2.78 (d, 1H, *J* = 18.7 Hz), 2.66 (dd, 1H, *J* = 19.0, 7.4 Hz), 2.57 (d, 1H, *J* = 18.7 Hz), 1.56–1.47 (m, 4H), 1.38 (sextet, 4H, *J* = 7.3 Hz), 1.20 (s, 3H), 0.86 (t, 6H, *J* = 7.3 Hz); ^13^C NMR (100 MHz, DMSO-*d*_6_): δ 175.10, 165.35, 165.31, 145.78, 138.79, 138.62, 109.41, 70.60, 69.34, 36.28, 34.64, 32.33, 30.31, 25.81, 21.62,13.89,; ESI-MS *m/z*: 465.2 [M − H]^−^; HRMS (EI) calcd for C_23_H_30_O_6_S_2_, 466.1484; found, 466.1483.

#### 3.5.3. 6,7-Bis(*n*-hexylthio) 1-Deoxy-6-demethoxybostrycin (**20**)

A red solid (MeOH) in a 55% yield; mp: 170–172 °C; IR (KBr): ν_max_ = 3322, 2956, 2925, 2853, 1599, 1439, 1422, 1408 cm^−1^; ^1^H NMR (400 MHz, DMSO-*d*_6_): δ 13.11 (s, 2H), 4.79 (br s, 1H), 4.45 (s, 1H), 3.62 (br t, 1H), 3.23 (t, 4H, *J* = 7.2 Hz), 2.82 (dd, 1H, *J* = 19.2, 5.4 Hz), 2.77 (d, 1H, *J* = 18.7 Hz), 2.65 (dd, 1H, *J* = 19.2, 7.3 Hz), 2.56 (d, 1H, *J* = 18.7 Hz), 1.57–1.47 (m, 4H), 1.40–1.33 (m, 4H), 1.27–1.21 (m, 8H), 1.20 (s, 3H), 0.87–0.80 (m, 6H); ^13^C NMR (100 MHz, DMSO-*d*_6_): δ 175.22, 165.19, 165.14, 145.83, 138.74, 138.59, 109.36,70.60, 69.32, 36.29, 34.97,31.19, 30.29, 30.20, 28.12, 25.81, 22.43, 14.28; ESI-MS *m/z*: 521.1 [M − H]^−^; HRMS (EI) calcd for C_27_H_38_O_6_S_2_, 522.2110; found, 522.2107.

#### 3.5.4. 6,7-(Ethan-1′,2′-yl-dithio) 1-Deoxy-6-demethoxybostrycin (**21**)

A red solid (MeOH) in a 47% yield; mp: 226–227 °C; IR (KBr): ν_max_ = 3529, 3479, 2974, 2922, 2849, 2824, 1588, 1515, 1449, 1415 cm^−1^; ^1^H NMR (400 MHz, DMSO-*d*_6_): δ 12.64 (s, 2H), 4.80 (d, 1H, *J* = 5.2 Hz), 4.45 (s, 1H), 3.64 (dt, 1H, *J* = 7.5, 5.2 Hz), 3.34 (s, 4H), 2.85 (dd, 1H, *J* = 18.9, 5.4 Hz), 2.80 (d, 1H, *J* = 18.4 Hz), 2.66 (dd, 1H, *J* = 18.9, 7.7 Hz), 2.59 (d, 1H, *J* = 18.4Hz), 1.21 (s, 3H); ^13^C NMR (100 MHz, DMSO-*d*_6_): δ 180.71, 156.93, 156.86, 140.79, 137.10, 136.93, 107.63, 107.60, 70.63, 69.29, 36.48, 30.33, 26.68, 25.89; ESI-MS *m/z*: 379.1 [M − H]^−^; HRMS (EI) calcd for C_17_H_16_O_6_S_2_, 380.0388; found, 380.0380.

#### 3.5.5. 6,7-(Butan-2′,3′-yl-dithio) 1-Deoxy-6-demethoxybostrycin (**22**)

A red solid (MeOH) in a 78% yield; mp: 225–227 °C; [α]^20^_D_ = −188.7° (*c* = 1.00, CH_3_OH); IR (KBr): ν_max_ = 3484, 3406, 2969, 2930, 2873, 2818, 1582, 1515, 1443, 1413 cm^−1^; ^1^H NMR (400 MHz, DMSO-*d*_6_): δ 12.68 (s, 1H), 12.66 (s, 1H), 4.79 (d, 1H, *J* = 5.2 Hz), 4.44 (s, 1H), 3.68–3.44 (m, 3H), 2.84 (dd, 1H, *J* = 18.6, 5.2 Hz), 2.80 (d, 1H, *J* =18.5 Hz), 2.66 (dd, 1H, *J* = 18.6, 7.7 Hz), 2.58 (d, 1H, *J* = 18.5 Hz), 1.32 and 1.30 (each d, 3H, *J* = 6.0 Hz), 1.21 (s, 3H); ^13^C NMR (100 MHz, DMSO-*d*_6_): δ 180.96, 180.86, 156.73, 156.65, 139.83, 139.29, 136.96, 136.84, 107.75, 70.59, 69.26, 36.48, 30.30, 25.90, 23.33, 18.08; ESI-MS *m/z*: 407.1 [M − H]^−^; HRMS (EI) calcd for C_19_H_20_O_6_S_2_, 408.0698; found, 408.0701.

### 3.6. Antitumor Activity *in Vitro*

#### 3.6.1. Cell Culture

MDA-MB-435, HepG2 and HCT-116 cells were cultured in Dulbecco’s modification Eagle’s medium (DMEM, Invitrogen, Carlsbad, CA, USA) supplemented with 10% fetal bovine serum (FBS, Hyclone, Logan, UT, USA), 2 mM L-glutamine, 100 μg/mL streptomycin and 100 U/mL penicillin (Invitrogen). The cells were incubated at 37 °C in a humidified atmosphere of 5% CO_2_.

#### 3.6.2. Assessment of Antitumor Activity by MTT Assay

Cells were seeded in 96-well flat-bottom plates at a density of 1 × 10^4^ cells/mL, and cultured in a humidified incubator (5% CO_2_) at 37 °C for 24 h, followed by exposure to various concentrations of compounds tested for 48 h. Subsequently, 20 μL of MTT reagent (Genview, Houston, TX, USA, 5 mg/mL) dissolved in PBS (pH 7.4) was added to each well and mixed, the cells were then incubated for an additional 4 h. Culture supernatant was moved, 150 μL of DMSO (Sangon Biotech, Shanghai, China) was added to each well to fully dissolve the MTT-formazan crystals. Cell growth inhibition was determined by measuring the absorbance (Abs) at λ = 570 nm using a microplate reader and calculated according to the following equation:

[Growth inhibition = (1 − OD of treated cells/OD of control cells) × 100%](1)

The half maximal inhibitory concentrations (IC_50_) were obtained from liner regression analysis of the concentration-response curves plotted for each tested compound.

## 4. Conclusions

In this paper, 21 derivatives of deoxybostrycin were designed, synthesized and evaluated for their anti-tumor activity against MDA-MB-435, HepG2 and HCT-116 cell lines. The bioassay results indicated that most of these derivatives possess good anti-tumor activities. The substitution pattern on the anthraquinone ring affected anticancer activity remarkably. It was conﬁrmed that a methoxyl at C-6 is not necessary for cytotoxic activity. However, acetonide formed at C-2, C-3 in compound **2** strongly reduces cytotoxic activity. Replacement of the methoxyl at C-6 with amines does not improve the anti-tumor activity. Introduction of alkylthio groups at C-6 and C-7 positions of the deoxybostrycin improved the cytotoxicity greatly. In particular, compounds **19**, **21** and **22** displayed the highest cellular cytotoxicity against MDA-MB-435 and distinguished themselves as potential anti-tumor agents.
